# Perivascular space diffusivity and brain microstructural measures are associated with circadian time and sleep quality

**DOI:** 10.1111/jsr.14226

**Published:** 2024-04-27

**Authors:** Kristoffer Brendstrup‐Brix, Sara Marie Ulv Larsen, Hong‐Hsi Lee, Gitte Moos Knudsen

**Affiliations:** ^1^ Neurobiology Research Unit Copenhagen University Hospital Rigshospitalet Copenhagen Denmark; ^2^ Faculty of Health and Medical Sciences University of Copenhagen Copenhagen Denmark; ^3^ Athinoula A. Martinos Center for Biomedical Imaging, Department of Radiology Massachusetts General Hospital Charlestown Massachusetts USA; ^4^ Harvard Medical School Boston Massachusetts USA

**Keywords:** circadian rhythms, cognitive function, glymphatic system, neuroimaging, sleep and the brain

## Abstract

The glymphatic system is centred around brain cerebrospinal fluid flow and is enhanced during sleep, and the synaptic homeostasis hypothesis proposes that sleep acts on brain microstructure by selective synaptic downscaling. While so far primarily studied in animals, we here examine in humans if brain diffusivity and microstructure is related to time of day, sleep quality and cognitive performance. We use diffusion weighted images from 916 young healthy individuals, aged between 22 and 37 years, collected as part of the Human Connectome Project to assess diffusion tensor image analysis along the perivascular space index, white matter fractional anisotropy, intra‐neurite volume fraction and extra‐neurite mean diffusivity. Next, we examine if these measures are associated with circadian time of acquisition, the Pittsburgh Sleep Quality Index (high scores correspond to low sleep quality) and age‐adjusted cognitive function total composite score. Consistent with expectations, we find that diffusion tensor image analysis along the perivascular space index and orbitofrontal grey matter extra‐neurite mean diffusivity are negatively and white matter fractional anisotropy positively correlated with circadian time. Further, we find that grey matter intra‐neurite volume fraction correlates positively with Pittsburgh Sleep Quality Index, and that this correlation is driven by sleep duration. Finally, we find positive correlations between grey matter intra‐neurite volume fraction and cognitive function total composite score, as well as negative interaction effects between cognitive function total composite score and Pittsburgh Sleep Quality Index on grey matter intra‐neurite volume fraction. Our findings propose that perivascular flow is under circadian control and that sleep downregulates the intra‐neurite volume in healthy adults with positive impact on cognitive function.

## 1 INTRODUCTION

The glymphatic system is a mechanism that regulates directional interstitial fluid movement and waste clearance by means of bulk movement of cerebrospinal fluid (CSF) from the subarachnoid space along periarterial spaces, where it mixes with interstitial fluid within the parenchyma before ultimately exiting from the parenchyma via perivenous spaces (Iliff et al., 2012). It is proposed as a restorative and neuroprotective mechanism tightly linked to sleep (Hablitz & Nedergaard, 2021); in mice, the brain interstitial space volume increases by ~65% during sleep relative to wakefulness (Xie et al., 2013), and the system is under circadian control with a peak volume at subjective night (Hablitz et al., 2020). In humans suspected of CSF disorders (i.e. CSF leakage, pineal cyst, arachnoid cyst, hydrocephalus and idiopathic intracranial hypertension), parenchymal clearance of an intrathecally administered contrast agent is reduced by acute (Eide et al., 2021) and chronic sleep deprivation (Eide et al., 2022). Unfortunately, the invasive nature of this method limits the ability to investigate brain clearance in healthy humans. Recent advances in non‐invasive techniques, particularly ultrafast magnetic resonance imaging (MRI) and diffusion weighted MRI (DWI), may provide measures of relevance for brain clearance. Diffusion tensor image analysis along the perivascular space (DTI‐ALPS) is a DWI‐based non‐invasive technique, which provides an index of CSF diffusion along the perivascular spaces in the periventricular white matter (Taoka et al., 2017). Although this sensitivity to perivascular space diffusivity suggests that the DTI‐ALPS index could provide a measure of activity of the glymphatic system in humans (Taoka et al., 2017), structural properties of the surrounding white matter might also be of importance. Since being introduced in 2017, the method has been applied in a wide range of diseases, including neurodegenerative diseases such as Alzheimer's and Parkinson's disease (Cai et al., 2023; Kamagata et al., 2022; Taoka et al., 2017). In these patients, DTI‐ALPS indices are often lower, which has been interpreted as an impairment of the glymphatic system (Taoka et al., 2017). In a cohort of healthy older adults, Siow and colleagues compared DTI‐ALPS with polysomnographic and neuropsychological measures, and found that non‐rapid eye movement stage 2 sleep duration was positively and apnea–hypopnea index (AHI) negatively associated with DTI‐ALPS indices, and that high DTI‐ALPS indices are associated with better memory and language test performance (Siow et al., 2022). In humans, the DTI‐ALPS index correlates positively with washout rate of an intrathecally administered MRI‐contrast agent (Zhang, Zhou, et al., 2021), and negatively with cerebral amyloid‐β (Aβ) and tau depositions, as measured with positron emission tomography (PET; Ota et al., 2022). In patients with Alzheimer's disease, DTI‐ALPS indices correlate positively with cognitive function as measured with the Mini‐Mental State Exam (MMSE; Kamagata et al., 2022; Taoka et al., 2017). Finally, the DTI‐ALPS index is positively correlated with Aβ42 in CSF, a biomarker of brain amyloidosis with low CSF levels linked to high accumulation of Aβ, as well as 18F‐Flourodeoxyglucose PET (FDG‐PET) uptake, a measure of cerebral glucose metabolism linked to neurodegeneration (Cohen et al., 2019; Kamagata et al., 2022). These findings support the notion of the DTI‐ALPS index as a measure of brain clearance.

Although the glymphatic system in animals has been tightly linked with sleep (Hablitz & Nedergaard, 2021) and that sleep deprivation reduces the clearance of an intrathecally administered contrast agent (Eide et al., 2021), sleep‐deprived individuals still clear the contrast only at a rate approximately 50% lower than when asleep. Given that brain clearance also takes place while awake, investigations of indices of brain clearance during day time can provide important information in themselves and in their relation to sleep quality and cognitive function.

While the glymphatic system hypothesis bridges the gap between sleep and neurodegenerative disease (Bubu et al., 2017; Lucey et al., 2018; Shokri‐Kojori et al., 2018), this mechanism does not yet fully explain the cognitive decline following acute sleep deprivation. Reflecting the multifaceted nature of sleep, multiple mechanisms within the brain might be at play. According to the synaptic homeostasis hypothesis, sleep acts as a synaptic downscaling mechanism, which counteracts synaptic strengthening occurring during wakefulness (Cirelli & Tononi, 2017). Given that continued potentiation would lead to saturation of brain neural networks, increasing energetic needs and low neural signal‐to‐noise ratio, the downscaling during sleep is crucial for continued learning when awake and hence cognitive function. In animals, it has been shown that wake increases the size of axon–spine interfaces and consequently synapse strength (Holtmaat & Svoboda, 2009; Nishiyama & Yasuda, 2015), and that this process is reversed by sleep (Cirelli & Tononi, 2017; de Vivo et al., 2017). Recently, this has been complemented by evidence of sleep–wake‐induced fluctuations in human brain structural measures, with increases during wakefulness in white matter fractional anisotropy (FA; Voldsbekk et al., 2020), grey matter tissue density and cortical thickness (Voldsbekk et al., 2022), as well as decreases in grey matter mean diffusivity (MD; Bernardi et al., 2016). Taken together, these findings point to increases in white matter integrity and grey matter dendritic density during wakefulness. However, although sensitive to differences on a microstructural scale, multiple biological processes could give rise to the observed sleep–wake‐related differences (Mori & Tournier, 2014). Here, we applied a microstructural model in order to estimate microscopic brain features unconfounded by the effects of crossing white matter fibres or neurite orientation dispersions (Kaden, Kelm, et al., 2016; Kaden, Kruggel, & Alexander, 2016). Using this model, we extracted measures of grey and white matter dendritic and axonal volume, measured collectively as the intra‐neurite volume fraction (INVF), as well as extra‐neurite mean diffusivity (exMD) arising from a space comprised of neurite somas, glial cells and extracellular space.

Here we hypothesized that: (1) DTI‐ALPS index correlates negatively with circadian time and positively with cognitive function as measured with the age‐adjusted cognitive function total composite (CFTC) score; (2) grey matter INVF correlates positively and exMD negatively with circadian time; (3) white matter FA correlates positively with circadian time; and (4) grey matter INVF correlates positively and exMD negatively with sleep quality as measured with the Pittsburgh Sleep Quality Index (PSQI) score (high scores correspond to low sleep quality).

## 2 METHODS

### 2.1 Study population

**FIGURE 1:**
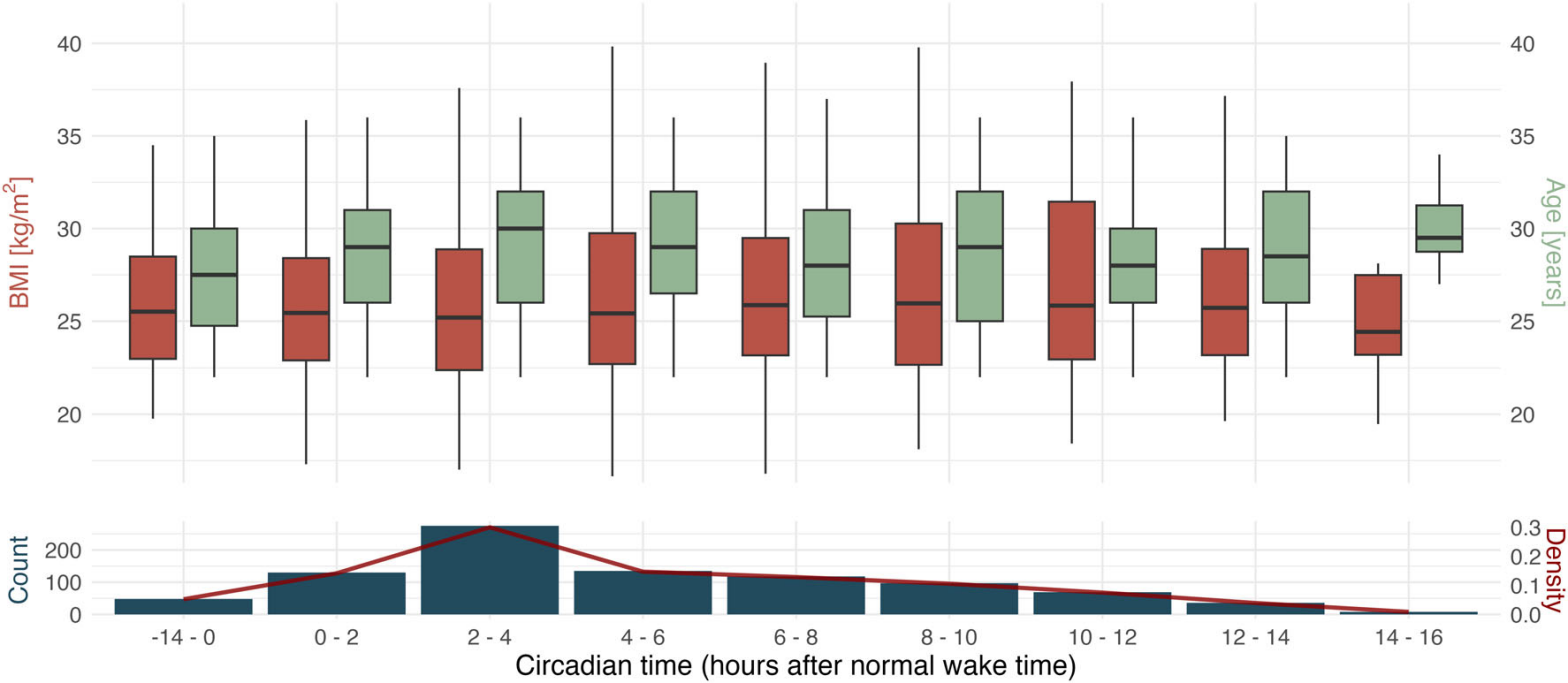
Binned circadian time of acquisition of diffusion weighted MRI (DWI) and corresponding distribution of BMI and age. Histogram showing subject count in each 2‐hr bin (and one bin covering all subjects with a calculated circadian time below zero, i.e. acquisition of DWI earlier than usual awakening time), and corresponding BMI and age distribution. BMI, body mass index; MRI, magnetic resonance imaging.

Data used in this study were extracted from the WU‐Minn Consortium of the Human Connectome Project (HCP; Van Essen et al., 2013; publicly available at https://www.humanconnectome.org/). This dataset contains DWIs from 1060 subjects collected with approximately even distribution from around 08:00 hours 21:00 hours (Figure 1). Participants had no history of significant cardiovascular, psychiatric or neurological disorders, no history of head injury, and had a score of >25 on MMSE (Folstein et al., 1975) on initial visit day. One subject was included in the HCP database despite scoring 23 on the MMSE. Because this subject was not an outlier in any of the analysed metrics, the subject was included in all analyses in the present study. A full list of inclusion and exclusion criteria can be found in Van Essen et al. (2013). Subjects with structural brain anomalies were assessed for eligibility, and excluded if the anomaly resulted in segmentation error (*n* = 14) or was suspected to potentially bias brain parenchymal microstructural measures (i.e. benign cysts, lacunes, cavernomas or developmental venous anomalies; *n* = 15). The cohort included both mono‐ (*n* = 272) and dizygotic (*n* = 153) twins as well as half and full siblings as described in the HCP S1200 release reference manual (available at https://db.humanconnectome.org/data/projects/HCP_1200). For monozygotic twins, only one twin was included in the analyses. The excluded twin was selected based on a low MMSE score (MMSE < 28; *n* = 8), acquisition of DWI earlier than usual awakening time (*n* = 2) or at random (*n* = 119). This left a total of 916 subjects (492 females) for analysis.

### 2.2 Preprocessing of DWIs

Spin Echo EPI DWIs were acquired on the second study day with a Siemens 32‐channel receive head coil on a Siemens Skyra 3 T MR scanner at University of Washington in St Louis, Missouri, USA. Images were acquired with the following parameters: TR = 5520 ms; TE = 89.5 ms; flip angle = 78°; refocusing flip angle = 160°; FoV = 210 × 180 mm^2^; matrix = 168 × 144; 111 slices with 1.25 mm slice thickness; 1.25 mm isotropic voxels; MB factor = 3; echo spacing = 0.78 ms; bandwidth = 1488 Hz/Px; partial Fourier = 6/8; *b*‐values = 1000, 2000 and 3000 s/mm^2^; and an acquisition time of 9 min and 50 s for each set. Images were collected in six sets, including right‐to‐left and left‐to‐right phase encoding direction pairs and the application of a different diffusion gradient table for each *b*‐value, with approximately 90 DW directions and six images with no DW. All images were extracted following preprocessing according to the HCP diffusion pipeline (v. 3.19.0) as described in the HCP minimal processing pipeline (Glasser et al., 2013). Given that: (i) subjects released as part of different release packages were re‐preprocessed using the same preprocessing pipeline; and (ii) evaluated measures did not depend on initial release package (Figure S1), all subjects were pooled in the same analyses.

### 2.3 Preprocessing of structural images

The 3D MPRAGE T1w images were acquired with the following parameters: TR = 2400 ms, TE = 2.14 ms, TI = 1000 ms, flip angle = 8°, FoV = 224 × 224 mm^2^, 0.7 mm isotropic voxels, bandwidth = 210 Hz/Px, iPAT = 2, and an acquisition time of 7 min and 40 s. All images were extracted following preprocessing according to the HCP pipeline (Glasser et al., 2013), including cortical reconstruction and volumetric segmentation with FreeSurfer (Fischl, 2012; version 5.3.0). Regions for regional analysis were as defined in the Desikan–Killiany atlas (Desikan et al., 2006), whereas grey and white matter regions of interest (ROIs) were defined as all cortical grey matter labelled in the FreeSurfer cortical reconstruction and as all cerebral white matter, respectively.

### 2.4 Modelling of DWI

We fitted diffusion tensors to the preprocessed DWIs of *b*‐value = 1000 s/mm^2^ with MRtrix tools “*dwi2tensor*” and “*tensor2metric*” (Tournier et al., 2019), which yielded maps of FA and MD. Additionally, multi‐compartment microstructural measures were calculated by applying the spherical mean technique (SMT; version 0.4; Kaden, Kruggel, & Alexander, 2016) to DWIs of all *b*‐values. This method allows for disentanglement of contributions to diffusivity arising from intra‐neurite (dendrites and axons) and extra‐neurite (neuronal somas, glial cells and extracellular space) water compartments without prior knowledge of fibre orientation distribution or intrinsic diffusivity (Kaden, Kelm, et al., 2016). This is done under the assumption of no effective transverse diffusivity in the neurite compartment, no difference in longitudinal microscopic diffusion inside and outside of neurites, and that transverse microscopic diffusivity in the extra‐neurite compartment can be expressed as a function of the INVF and intrinsic diffusivity (tortuosity relation). After separating intra‐ and extra‐neurite water pools, the INVF is defined as the ratio of axonal and dendritic water volume to the overall volume of water in both intra‐ and extra‐neurite spaces, whereas the exMD is defined as the MD in the extra‐neurite space (Kaden, Kelm, et al., 2016). The SMT has previously been applied to the present HCP dataset demonstrating good consistency and reproducibility in estimated microstructural measures (Kaden, Kelm, et al., 2016). All data were fitted in subject space, and average values of INVF and exMD were extracted per ROI.

### 2.5 Calculation of DTI‐ALPS index

**FIGURE 2:**
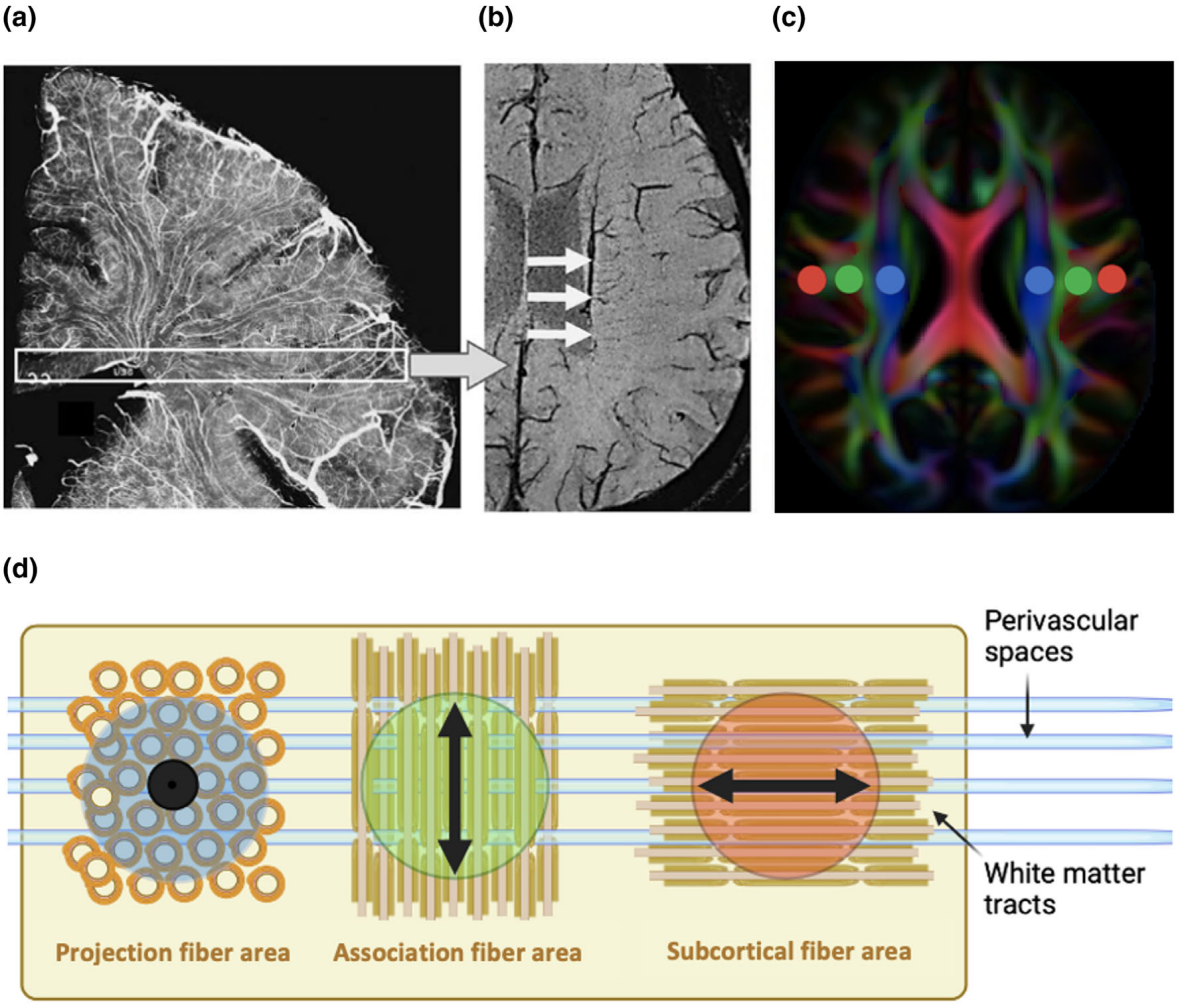
Concept for the diffusion tensor image analysis along the perivascular space (DTI‐ALPS) method. (a, b) Borrowed with permission from Taoka et al. (2017). (a) Roentgenogram of an injected coronal brain slice showing parenchymal vessels that run horizontally on the slice (white box) at the level of the lateral ventricle body. (b) Axial on the slice at the level of the lateral ventricle body indicates that parenchymal vessels run laterally (*x*‐axis). (c) Colour display of DTI in MNI152 standard space indicating the distribution of projection fibres (*z*‐axis: blue), association fibres (*y*‐axis: green) and the subcortical fibres (*x*‐axis: red). Three ROIs are placed bilaterally in the area with projection fibres, association fibres and subcortical fibres. (d) Schematic indicating the relationship between the direction of the perivascular space (blue cylinders) and the directions of the fibres. Note that the direction of the perivascular space is perpendicular to both projection and association fibres (created with BioRender.com). DTI, diffusion tensor imaging; ROI, region of interest; SWI, susceptibility weighted MRI.

The DTI‐ALPS indices were calculated as described by Taoka et al. (2017). Exploiting the fact that perivascular spaces surrounding the medullary veins bypass white matter rich in first association fibres (principally oriented anterior‐to‐posterior, that is, perpendicular to the perivascular spaces) and then projection fibres (principally oriented head‐to‐foot, also perpendicular to the perivascular spaces), an index sensitive to differences in diffusivity along and transverse to the perivascular space can be calculated (Figure 2). In MNI152 standard‐space, two periventricular white matter regions rich in projection and association fibres, respectively, were drawn on both hemispheres using a HCP1065 standard‐space DTI template RGB‐map for guidance. For each subject, the regions were then moved into subject space using transformation matrices previously calculated from the structural data and used to mask the estimated tensor. The mean sizes of the ROIs in subject space were 231.1 mm^3^ (SD = 45.0) and 282.1 mm^3^ (SD = 46.6) for association and projection fibre areas, respectively. Averaging across non‐zero voxels then yielded estimated projection (*D*_proj_) and association fibre (*D*_assoc_) tensor values, which were used for calculation of the DTI‐ALPS index for each individual as follows, with subscripts *x*, *y* and *z* indicating diffusivity in the corresponding direction in space: DTI‐ALPS index = mean(*D*_xproj_, *D*_xassoc_)/mean (*D*_yproj_, *D*_zassoc_). DTI‐ALPS indices were calculated independently on both hemispheres and averaged, yielding one index per subject. All transformations were visually inspected to ensure accurate and precise demarcations of the corresponding fibre areas.

### 2.6 Behavioural data

On the first study day, all subjects underwent assessment of subjective sleep quality as measured with the PSQI (Buysse et al., 1989) as well as extensive neuropsychological testing, which was summarized in the combined variable CFTC. The CFTC score is an age‐adjusted score combining test scores from the NIH Toolbox Assessment of Neurological and Behavioural function, which has been normalized to 100 with scores of 85 and 115 describing performance 1 SD below and above age average, respectively (Akshoomoff et al., 2014). Included in the CFTC are measures of fluid (i.e. executive function, episodic memory, working memory and processing speed) and crystallized (i.e. vocabulary comprehension and reading decoding) cognitive measures. The PSQI contains seven composite scores, which can be combined into one global score ranging from 0 (highest self‐reported sleep quality) to 21 (lowest self‐reported sleep quality), with cut‐off values of 5–8 showing good sensitivity and specificity in separating good and poor sleepers (Buysse et al., 1989; Fabbri et al., 2021). Circadian time of acquisition is here defined as time from self‐reported usual awakening time to mid‐scan time.

### 2.7 Statistics

All statistics were computed using R (RStudio Team [2022]. RStudio: Integrated Development Environment for R. RStudio, PBC, Boston, MA, USA). Statistical tests for correlation were computed as Pearson correlation coefficients, group differences were tested with Student's *t*‐tests, and linear modelling was performed using the R package “*stats*” (version 4.2.1). All measures included in statistical analyses were normal distributed on visual inspection. Regional analysis outcomes were corrected for multiple comparisons with a Bonferroni correction (*p*_FWER_; *α* = 0.05).

## 3 RESULTS

Subject characteristics are summarized in Table 1. Data were acquired at a circadian time ranging from −13.3 to 14.9 hr, equivalent to acquisition 13.3 hr prior to and 14.9 hr after usual awakening time, respectively. In general, the cohort showed high scores on cognitive tests, with an average CFTC score of 113.6 equivalent to almost 1 SD above age average (Akshoomoff et al., 2014).

**TABLE 1:** Cohort characteristics.

Variable	Mean ± SD (range)
Age (years)	28.7 ± 3.7 (22; 37)
BMI (kg m^−2^)	26.5 ± 5.23 (16.7; 47.8)
Systolic/diastolic blood pressure (mmHg)	123.5/76.5 ± 13.8/10.5 (87/48; 185/115)
Years of school (years)	14.9 ± 1.78 (11; 17)
Acquisition time (HH:MM)	12:04 hours ± 3:19 (07:31 hours; 21:32 hours)
Usual awakening time (HH:MM)	07:04 hours ± 2:04 (01:15 hours; 22:00 hours)
Circadian time of acquisition (hr)	5.0 ± 3.9 (−13.3; 14.9)
PSQI score	4.85 ± 2.80 (0; 19)
Usual amount of sleep per night (hr)	6.80 ± 1.15 (2.5; 11)
Age‐adjusted CFTC score	113.6 ± 20.19 (59.34; 153.4)
MMSE score	29.0 ± 1.03 (23; 30)

*Note*: The cohort had a prevalence of obesity (defined as BMI > 30 kg m^−2^) of 22%, and of severe obesity (defined as BMI > 40 kg m^−2^) of 2.1%. 27% of the subjects had hypertension defined as a systolic blood pressure above 135 mmHg and/or a diastolic blood pressure above 85 mmHg.

Abbreviations: BMI, body mass index; CFTC, cognitive function total composite; MMSE, Mini‐Mental State Exam; PSQI, Pittsburgh Sleep Quality Index.

### 3.1 DTI‐ALPS index correlates with circadian time

**FIGURE 3:**
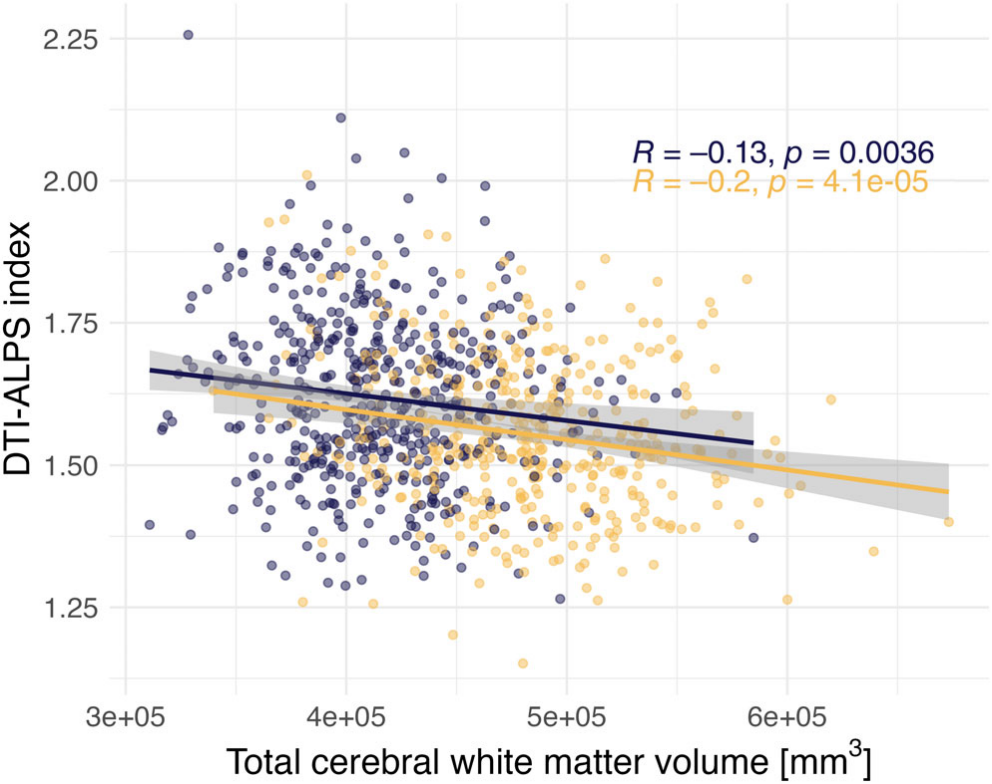
Diffusion tensor image analysis along the perivascular space (DTI‐ALPS) relative to brain structural and microstructural measures. DTI‐ALPS index correlates negatively with total cerebral white matter volume (yellow points represent male sex and purple points female sex). Correlations are tested with Pearson correlation tests. 95% CI is shown in grey shading. CI, confidence interval.

The DTI‐ALPS index correlates negatively with circadian time after correction for total cerebral white matter volume, sex, age, systolic blood pressure and body mass index (BMI; *β* = −0.070, *p* = 0.03). This correlation was not significant after exclusion of subjects with a calculated circadian time below zero (i.e. subjects with MR acquisition earlier than usual awakening time; *n* = 48; *β* = −0.061, *p* = 0.07). Our model revealed highly significant inverse correlations between total white matter volume and DTI‐ALPS index (*β* = −0.198, *p* < 0.001; Figure 3). Male sex had a negative effect on DTI‐ALPS index (*β* = −0.101, *p* = 0.02), whereas the model revealed no effects of age, BMI or systolic blood pressure (age: *β* = −0.089, *p* = 0.77; BMI: *β* = 0.024, *p* = 0.49; systolic blood pressure: *β* = −0.077, *p* = 0.77; systolic blood pressure × age: *β* = −0.090, *p* = 0.81). Volume of the fibre ROIs in subject space did not correlate with DTI‐ALPS index (mean fibre ROI: *β* = 0.058, *p* = 0.28).

### 3.2 Brain microstructural measures correlate with circadian time

**FIGURE 4:**
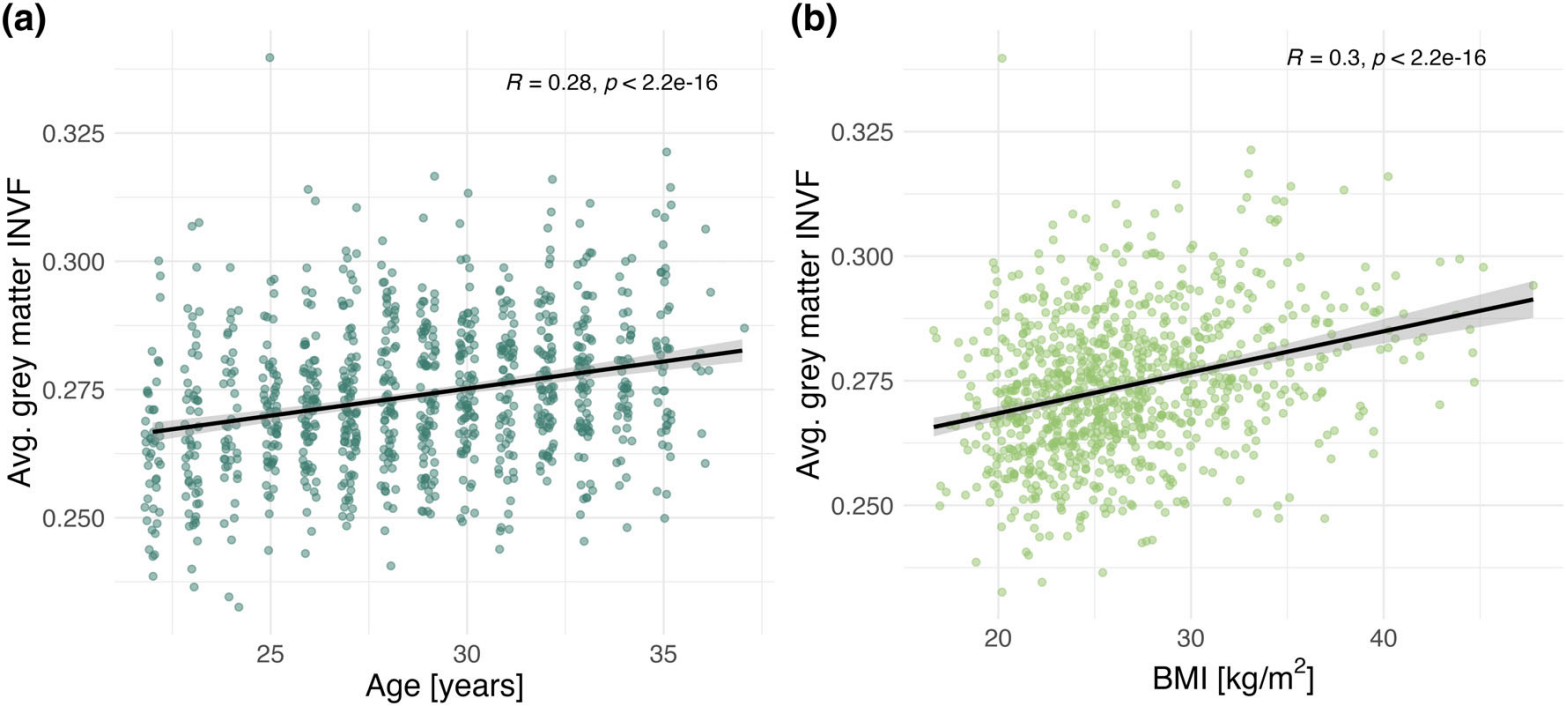
Average grey matter intra‐neurite volume fraction (INVF) relative to age and BMI. Average grey matter INVF relative to age (a) and BMI (b). Horizontal jitter was added in (a) in order to visualize overlaying datapoints. Correlations are tested with Pearson correlation tests. 95% CI is shown in grey shading. BMI, body mass index; CI, confidence interval.

Average white matter FA correlated positively with circadian time across all subjects (Pearson's *r* = 0.070, *p* = 0.03) and after exclusion of subjects with a negative circadian time of acquisition (*n* = 48; Pearson's *r* = 0.068, *p* = 0.04; Figure S2). White matter FA was found to correlate negatively with total cerebral white matter volume (Pearson's *r* = −0.143, *p* < 0.001), but correcting for total cerebral white matter volume did not significantly alter the correlation between average white matter FA and circadian time (*β* = 0.068, *p* = 0.04). Neither average white matter INVF nor exMD correlated with circadian time (INVF: *β* = 0.022, *p* = 0.51; exMD: *β* = −0.021, *p* = 0.52). Additionally, neither average grey matter INVF (Pearson's *r* = 0.011, *p* = 0.75) nor average grey matter exMD (Pearson's *r* = −0.046, *p* = 0.16) were correlated with circadian time. Average grey matter INVF was found to increase with age (Pearson's *r* = 0.28, *p* < 0.001; Figures 4a and S3) and BMI (Pearson's *r* = 0.3, *p* < 0.001; Figure 4b), but including age and BMI as covariates in a linear model did not reveal any correlation between grey matter INVF and circadian time (*β* = −0.024, *p* = 0.44). Regional analysis across all 34 regions in the Desikan–Killiany atlas revealed significant correlations between circadian time and exMD in medial (Pearson's *r* = −0.132, *p*_FWER_ = 0.002) and lateral orbitofrontal cortex (Pearson's *r* = −0.113, *p*_FWER_ = 0.02), while exMD in occipital, temporal and parietal regions did not correlate with circadian time (Figure S4b; Table S1). For INVF, no regions correlated significantly with circadian time after correcting for age, PSQI score and BMI (Figure S4a; Table S1).

### 3.3 Brain microstructural measures correlate with quality of sleep

**FIGURE 5:**
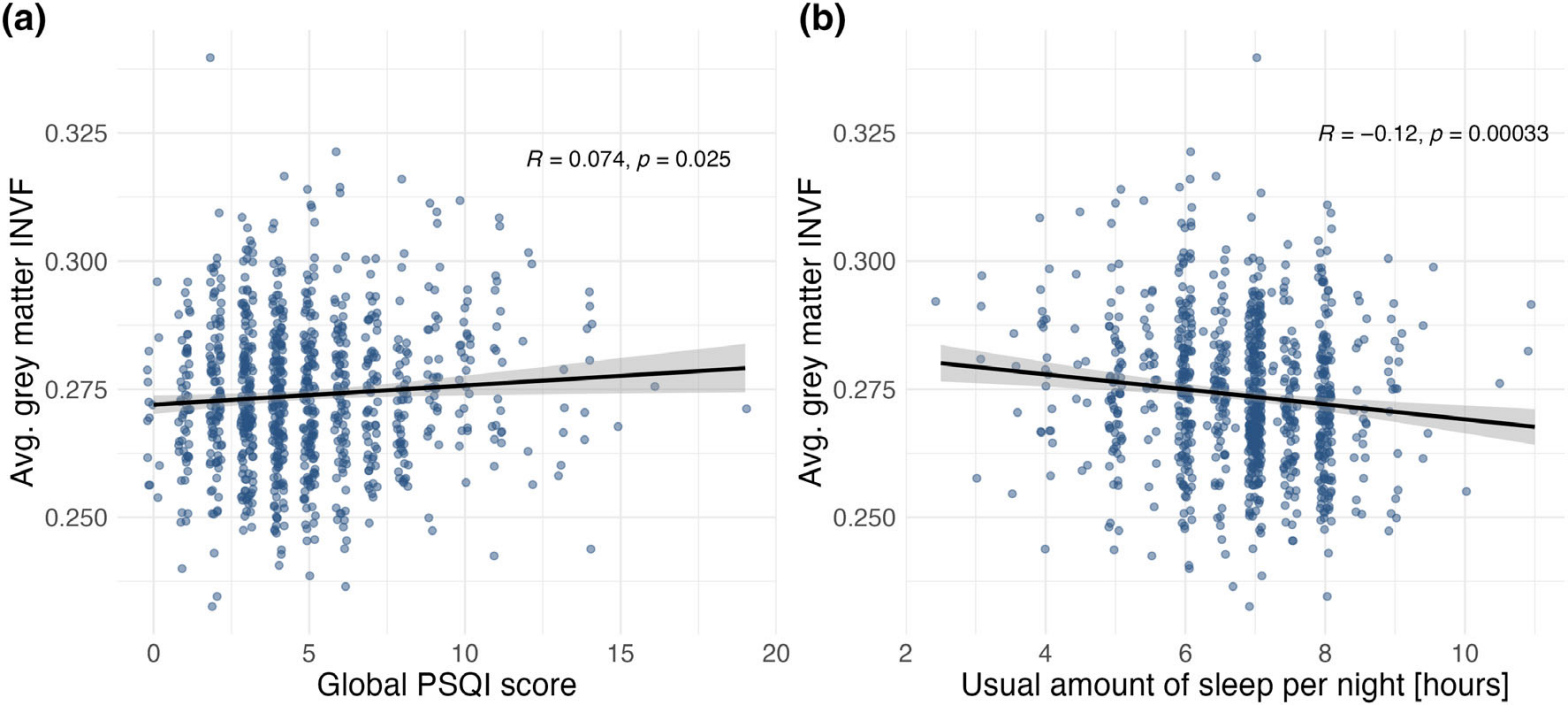
Average grey matter intra‐neurite volume fraction (INVF) relative to sleep quality and quantity. (a) Average grey matter INVF relative to global Pittsburgh Sleep Quality Index (PSQI) score. (b) Average grey matter INVF relative to usual amount of sleep per night in the last 7 days before acquisition. Horizontal jitter was added in order to visualize overlaying datapoints. Correlations are tested with Pearson correlation tests. 95% CI is shown in grey shading. CI, confidence interval.

**FIGURE 6:**
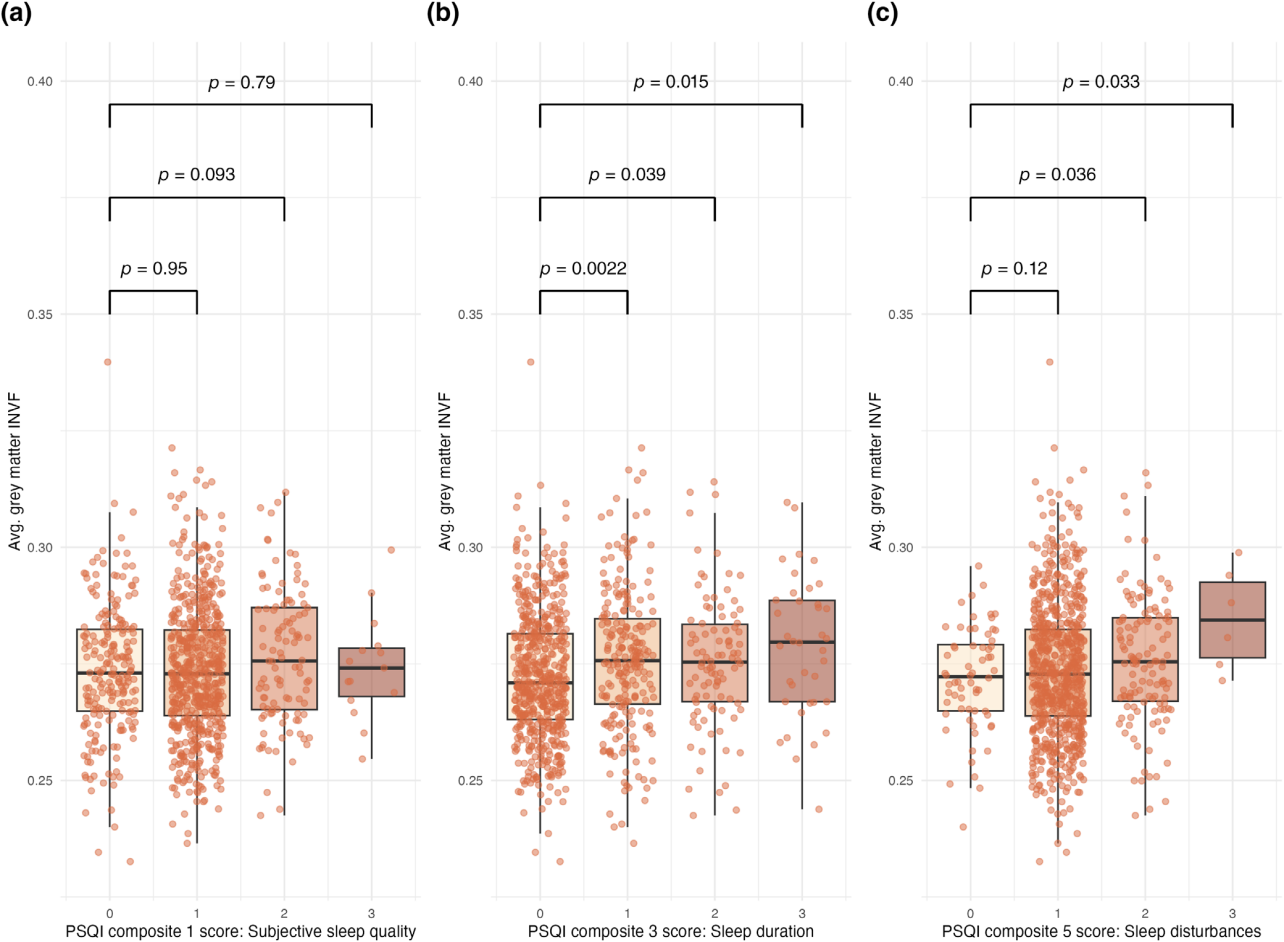
Average grey matter intra‐neurite volume fraction (INVF) relative to Pittsburgh Sleep Quality Index (PSQI) composite scores without correction for BMI and age. Average grey matter INVF relative to PSQI composites 1, 3 and 5 as defined in Buysse et al. (1989). Significance is tested with two‐tailed, unpaired Student's *t*‐tests (uncorrected *p*‐values). The upper and lower edge of the box represent first and third quartiles, the median value is represented by the line within the box, and whiskers extend from the edge of the box to 1.5 times the interquartile range. BMI, body mass index.

Average grey matter INVF correlated positively with global PSQI score (Pearson's *r* = 0.074, *p* = 0.025; Figure 5a). Subjects who reported to have significantly impaired sleep quality (PSQI >6) had higher average grey matter INVF than those with no or only slightly impaired sleep quality (PSQI ≤ 6; two‐sided, unpaired Student's *t*‐test: *t* = −2.27, *p* = 0.024). This trend remained after correcting for age and BMI (*β* = 0.045, *p* = 0.14). A linear model including all PSQI composites as well as age and BMI revealed that the correlation between global PSQI score and INVF was primarily driven by sleep duration (PSQI composite 3; *β* = 0.103, *p* = 0.004; Figure 6). Self‐reported usual amount of sleep per night was negatively correlated with average grey matter INVF (Pearson's *r* = 0.12, *p* < 0.001; Figure 5b), which remained significant after correction for BMI and age (*β* = 0.071, *p* = 0.02). Neither subjective sleep quality (PSQI composite 1; Figure 6a), sleep disturbances (PSQI composite 5; Figure 6c), sleep latency, habitual sleep efficiency, use of sleep medications nor daytime dysfunction were significantly related to INVF (PSQI composites 2, 4, 6 and 7; Figure S5). Similarly, average grey matter exMD (Pearson's *r* = −0.028, *p* = 0.39) and DTI‐ALPS index (Pearson's *r* = 0.008, *p* = 0.80; after correcting for total white matter volume, circadian time and sex: *β* = 0.014, *p* = 0.69) did not show any correlation with global PSQI score. Global PSQI score did not correlate with circadian time (Pearson's *r* = −0.043, *p* = 0.19; Figure S6a).

### 3.4 Cognitive measures relative to DTI‐ALPS index and brain microstructure

**FIGURE 7:**
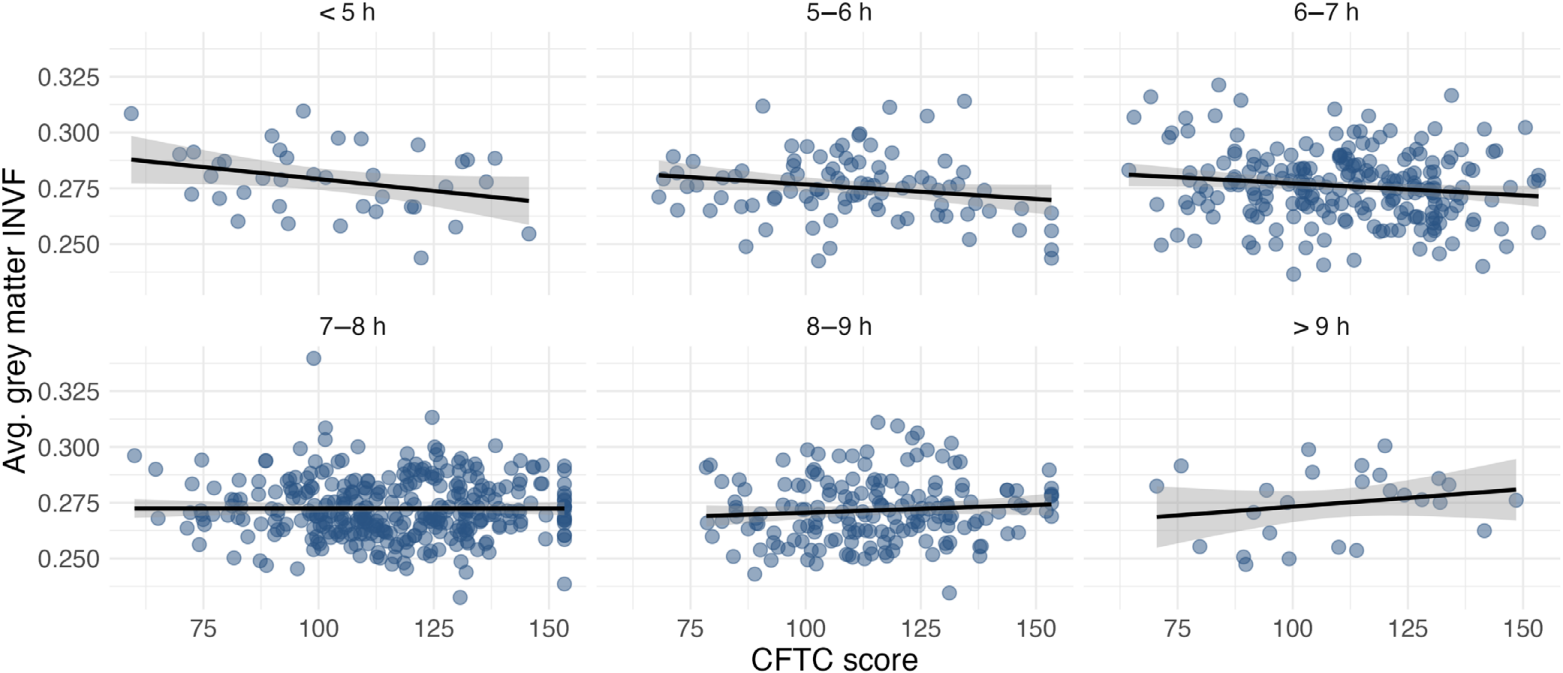
Age‐adjusted cognitive function total composite (CFTC) score relative to average grey matter intra‐neurite volume fraction (INVF) grouped according to usual sleep duration. In optimal sleep conditions (i.e. Pittsburgh Sleep Quality Index [PSQI] = 0 and sleep duration >8 hr), average grey matter INVF correlates positively with age‐adjusted CFTC scores (*β* = 0.12, *p* = 0.007). When subjective sleep quality decreases (i.e. higher PSQI and lower sleep duration), this correlation turns negative (usual sleep duration × age‐adjusted CFTC score: *β* = 0.97, *p* < 0.001). 95% CI is shown in grey shading. CI, confidence interval.

The CFTC score was found to correlate negatively with global PSQI score (Pearson's *r* = −0.12, *p* < 0.001; Figure S7), which led us to include global PSQI score, age and BMI as covariates in a linear model correlating average grey matter INVF with CFTC score. This model revealed a significant positive correlation between CFTC score and average grey matter INVF when regressing out the effect of age, BMI and PSQI (*β* = 0.149, *p* = 0.01), as well as significant negative interaction effects of PSQI score and CFTC score (*β* = −0.52, *p* = 0.002). Because usual sleep duration was the driver of the correlation between global PSQI score and average grey matter INVF, a separate model with self‐reported sleep duration (relative to 8 hr of sleep per night) in place of global PSQI score was created. This model showed a highly significant positive interaction effect of usual sleep duration and CFTC score on average grey matter INVF (*β* = 0.97, *p* < 0.001; Figure 7), as well as a significant positive correlation between average grey matter INVF and CFTC score (*β* = 0.12, *p* = 0.007). None of the cognitive subdomains included in the CFTC score (i.e. inhibition control and attention, cognitive flexibility, processing speed, language, episodic memory and working memory) or any of the two subtotal composites (fluid and crystallized cognition) deviated from or singlehandedly drove the observed correlations (data not shown). We found no significant correlation between CFTC score and DTI‐ALPS index (Pearson's *r* = −0.02, *p* = 0.49; after correcting for white mater volume, circadian time and sex: *β* = 0.026, *p* = 0.45) or average grey matter exMD (Pearson's *r* = 0.035, *p* = 0.29). Average white matter FA did not correlate with CFTC score (Pearson's *r* = 0.046, *p* = 0.16). CFTC score was not correlated with circadian time of acquisition (Pearson's *r* = −0.047, *p* = 0.16; Figure S6b).

## 4 DISCUSSION

### 4.1 DTI‐ALPS index correlates with circadian time

We find that in healthy young adults, diffusion along the perivascular space, as measured with the DTI‐ALPS index, correlates negatively with circadian time after correction for total white matter volume and sex. This finding points to circadian regulation of glymphatic flow during wakefulness, with lowered activity of the glymphatic system throughout the day. Previous evidence of circadian regulation of the glymphatic system is sparse. In rodents, interstitial space volume has been shown to be under circadian control with peak activity at night independent of sleep (Cai et al., 2020; Hablitz et al., 2020). Only one other study has previously investigated time‐of‐day effects on DTI‐ALPS. In this study on 84 older adults (Siow et al., 2022), they find no group differences in DTI‐ALPS indices when grouping according to MRI‐acquisition in the morning (09:00 hours to 12:00 hours) and evening (12:00 hours to 17:00 hours). This discrepancy might arise from several differences between the two study designs, including large differences in power and different age groups.

### 4.2 White matter volume and DTI‐ALPS index

When accounting for effects of circadian time and sex, we find that DTI‐ALPS index correlates negatively with total cerebral white matter volume. The cohort of young and healthy adults used in this study constitutes an ideal cohort for investigating such a relationship, as the presence of brain pathology assumably is negligible. Therefore, our findings point to white matter volume as a potential confounder of the DTI‐ALPS index. In one study separately reporting perivascular and white matter fibre contributions to the DTI‐ALPS index in patients with idiopathic normal pressure hydrocephalus, the lower DTI‐ALPS indices in these patients relative to healthy controls are primarily driven by increases in diffusivity orthogonal to both perivascular spaces and white matter fibres (Yokota et al., 2019). Additionally, this study finds a significant negative correlation between ventricular volume and DTI‐ALPS index. Taken together, these findings point to white matter volume and atrophy as a potential confounder of the DTI‐ALPS index. Although the DTI‐ALPS index has previously been found to correlate with the washout rate of an intrathecally administered MRI‐contrast agent in patients with cerebral small vessel disease (Zhang, Zhou, et al., 2021), chronic parenchymal changes in these patients might be a common confounder to both these measures. Ideally, future studies should validate the DTI‐ALPS index against more direct measures of glymphatic function, for example, contrast‐enhanced MRI or PET, in a healthy population.

### 4.3 Demographics influence on DTI‐ALPS index

Similar to previous studies, we find that DTI‐ALPS indices are lower in males (Siow et al., 2022; Zhang, Zhang, et al., 2021), but we find no effect of age, BMI or systolic blood pressure. Previous studies have found lower DTI‐ALPS indices with age (Cai et al., 2023; Taoka et al., 2022), although other similar studies report no age‐effect (Lee et al., 2022). The majority of these studies investigate older adults (60–90 years old), which could indicate that the age difference in DTI‐ALPS index is attributable to age‐related glymphatic dysfunction or brain atrophy. One study using DWI for ALPS reports age differences in a cohort of participants aged 15 to +80 years (Taoka et al., 2022), but finds no group differences in DWI‐ALPS indices in the age groups 10–19, 20–29 or 30–39 years. High systolic blood pressure as well as high pulse pressure has previously been linked with lower DTI‐ALPS indices in subjects aged 65–84 years (Kikuta et al., 2022). In the present study on young adults, we find no direct effect of systolic blood pressure or interaction effects between systolic blood pressure and age. Together with previous findings using DTI‐ALPS, our results point to a certain level of resilience of the glymphatic system in young adults.

### 4.4 Brain microstructural measures correlate with circadian time

We find that average white matter FA correlates positively with circadian time. This finding adds to the growing body of evidence for general increases in white matter integrity with wakefulness (Voldsbekk et al., 2020). Additionally, we find that medial and lateral orbitofrontal cortex exMD correlate negatively with circadian time. No regional or whole‐brain correlations between INVF and circadian time were found in this study. Because exMD encompasses MD across neuronal somas, glial cells and extracellular space, a decrease in diffusivity with circadian time in any of these compartments could explain the observed differences. Several studies point to regional differences in susceptibility to sleep deprivation, with prefrontal cortex being most susceptible (Eide et al., 2021; Harrison & Horne, 2000). Our inability to detect circadian fluctuations in exMD outside of the orbitofrontal cortex could be due to the absence of such fluctuations or because of much smaller circadian effects in these regions. Therefore, our findings suggest a microstructural foundation for prefrontal susceptibility to sleep deprivation.

### 4.5 Brain microstructural measures and age

The correlation between age and grey matter INVF in young adults points to continued myeloarchitectural remodelling in early adulthood, which is in line with previous studies (Kwon et al., 2018). We find this effect to be predominant in frontotemporal regions (Figure S3), which is in support of a protracted myelination in cortical areas involved in higher‐order cognitive functions into adulthood (Kwon et al., 2018).

### 4.6 Brain microstructural measures and BMI

Emerging evidence supports a relationship between body weight and sleep disturbances in young adults (Beccuti & Pannain, 2011). This relationship can in part be explained by an increase in risk of obstructive sleep apnea (OSA) in obese individuals (Jehan et al., 2017), although obesity appears to be associated with marked sleep disturbances and increased AHI independent of OSA (Beccuti & Pannain, 2011). Because the PSQI is not sensitive to objective sleep measures such as AHI (Fabbri et al., 2021), we speculate that the observed relationship between BMI and grey matter INVF is mediated by an increase in AHI with increasing BMI. Further studies are needed in order to clarify the effects of obesity and OSA on neurite density.

### 4.7 Brain microstructural measures correlate with usual sleep quality

We find average grey matter INVF to correlate positively with PSQI score. Subjects with a global PSQI score above 6 had higher INVF than those with scores below or equal to 6. In a linear model including age and BMI, this relationship was shown to be driven by usual sleep duration. Although related to sleep diary information and psychological sleep ratings, the PSQI global score has been shown not to correlate with objective sleep measures (Fabbri et al., 2021). Besides sleep duration, we find no significant effect of any other sleep characteristic on grey matter INVF (i.e. sleep disturbances, subjective sleep quality, sleep latency, habitual sleep efficiency, use of sleep medications or daytime dysfunction), which is in line with previous findings showing that sleep quantity and quality are related but orthogonal measures (Fabbri et al., 2021). This finding points to sleep as a downregulator of intra‐neurite volume.

### 4.8 Brain microstructural measure correlates with cognitive scores

We find that grey matter INVF correlates positively with CFTC score when accounting for age, BMI and PSQI score. Importantly, we find negative interaction effects between PSQI and CFTC score on INVF. Taken together, these findings point to two distinct biological mechanisms related to intra‐neurite volume. In optimal sleep conditions (i.e. low PSQI scores), a high intra‐neurite volume is related to high cognitive performance, which makes neurite density a positive contributor to cognitive performance. This role of neurite density is supported by evidence from normal aging and neurodegenerative disease, where reductions in synapse density correlate negatively with cognitive performance (Gozdas et al., 2021; Mecca et al., 2022). In suboptimal sleep conditions (i.e. high PSQI scores), a high intra‐neurite volume is related to low cognitive performance, which makes neurite density a marker with negative impact on cognitive performance. This paradoxical relationship is in line with the synaptic homeostasis hypothesis, as an increase in neurite density in the case of poor sleep quality is a reflection of an inability to properly downregulate synaptic density (Cirelli & Tononi, 2017). Improper synaptic renormalization will result in a low neural signal‐to‐noise ratio and saturation of plasticity, which in turn lowers cognitive performance. Therefore, our findings support the role of sleep as a downregulator of neurite density, and highlight the complexity in regulating the microstructural environment.

### 4.9 Limitations

Several limitations to the study should be noted. First, because only a minority of subjects underwent two MRI‐sessions, we could only analyse data from one timepoint per individual, which makes it harder to determine any causality. Second, when calculating circadian time of acquisition, we did so on the basis of self‐reported usual awakening times. These times, however, might be inaccurately reported or might not reflect the actual circadian rhythm (e.g. recent change in sleep–wake rhythmicity or recent travel across multiple time‐zones). Optimally, circadian phase at the time of acquisition is determined on the basis of body temperature, rest–activity cycles or melatonin levels. Third, both circadian and homeostatic effects might have contributed to the observed effects. In order to disentangle these effects, a forced desynchrony study design is needed. Fourth, we were only able to include subjective measures of sleep quality in our analyses. Although these measures are not related to those obtained with objective methods, they still provide valuable and reliable information of subjective sleep quality. Fifth, no measures were taken to ensure that individuals did not fall asleep during the MRI‐acquisition. Because brain clearance is thought to be different in sleep and wakefulness, sleep during MRI‐acquisition could bias our measures. Sixth, neuropsychological testing and acquisition of DWI were completed on separate study days. Although this distance in time could introduce bias, neuropsychological scores derived from the NIH toolbox have been shown to have strong test–retest reliability when tested twice within 7–21 days (Weintraub et al., 2013). Additionally, we find no significant effect of circadian time on CFTC scores (Figure S6b). Finally, both the DTI‐ALPS index and the microstructural measures used in analyses were derived from biophysical modelling of DWI. The evaluation of glymphatic flow through the DTI‐ALPS index in the brain white matter relies on the assumption of continuous perivascular spaces between white matter and cortex (Ringstad, 2024). However, most cortical vessels have no direct connections with vessels in the underlying white matter (Duvernoy et al., 1981). And, although the SMT model has been tested against histological samples with good consistency and reproducibility (Kaden, Kelm, et al., 2016), the model‐derived parameters, which are estimated under multiple assumptions, are indirect measures of microstructure and should be interpreted with caution (Novikov et al., 2018). Therefore, the in vivo evaluation of complicated grey and white matter microstructure may require more advanced imaging protocols and modelling techniques (Lee et al., 2020).

## 5 CONCLUSION

In this large study on young adults, we find diffusion along the perivascular space, white matter FA and orbitofrontal exMD to be under circadian regulation during wakefulness. These findings support that the glymphatic flow in healthy adults decreases with time awake, and proposes a microstructural foundation for prefrontal susceptibility to sleep deprivation. Additionally, low subjective sleep quality was associated with high INVFs, which links sleep quality to brain microstructure in healthy adults. Taken together, our study outcome supports the notion of the presence of a glymphatic system in humans, in addition to providing human evidence of sleep as a downregulator of neurite density.

## AUTHOR CONTRIBUTIONS

**Kristoffer Brendstrup‐Brix:** Conceptualization; formal analysis; methodology; writing – original draft; writing – review and editing; visualization. **Sara Marie Ulv Larsen:** Supervision; writing – review and editing; writing – original draft. **Hong‐Hsi Lee:** Methodology; writing – review and editing; formal analysis. **Gitte Moos Knudsen:** Conceptualization; supervision; writing – review and editing; writing – original draft.

## FUNDING INFORMATION

KBB was funded by the Danish Research Council (grant no. 10.46540/2061‐00041B; https://dff.dk/en) and by Rigshospitalet (grant no. R254‐A11214; https://www.rigshospitalet.dk). SMUL was funded by the Danish Research Council (grant no. 0134‐00453A). HHL was funded by the Office of the Director (OD) of the National Institutes of Health (NIH) and National Institute of Dental & Craniofacial Research (NIDCR) of NIH (grant no. DP5OD031854; https://www.nidcr.nih.gov). The funders had no role in study design, data collection and analysis, decision to publish, or preparation of the manuscript.

## CONFLICT OF INTEREST STATEMENT

The authors have declared that no conflict of interest exists.

## STUDY APPROVAL

Original data collection was approved by the Washington University in St. Louis Institutional Review Board (IRB ID #201204036). Written informed consent was acquired prior to study participation.

## Supporting information


**FIGURE S1.** Measures included in the analysis relative to release package. (a) Diffusion tensor image analysis along the perivascular space (DTI‐ALPS) index. (b) Average grey matter intra‐neurite volume fraction (INVF). (c) Average grey matter extra‐neurite mean diffusivity (exMD). (d) Average white matter fractional anisotropy (FA). The upper and lower edge of the box represent first and third quartiles, the median value is represented by the line within the box, and whiskers extend from the edge of the box to 1.5 times the interquartile range.
**FIGURE S2.** Average white matter fractional anisotropy (FA) relative to circadian time. Average white matter FA relative to circadian time across subjects with a calculated circadian time above zero. Correlation is tested with a Pearson correlation test.
**FIGURE S3.** Per region Pearson correlation coefficients between subject age and intra‐neurite volume fraction (INVF).
**FIGURE S4.** Regional microstructural measures relative to circadian time. (a) Per region *β* coefficients from a linear model correlating average grey matter intra‐neurite volume fraction (INVF) with circadian time of acquisition after correction for BMI, age and Pittsburgh Sleep Quality Index score. (b) Per region Pearson correlation coefficients between circadian time of acquisition and extra‐neurite mean diffusivity (exMD). Corresponding statistics can be found in Table S1.
**FIGURE S5.** Average grey matter intra‐neurite volume fraction (INVF) relative to Pittsburgh Sleep Quality Index (PSQI) composite scores without correction for BMI and age. Average grey matter INVF relative to PSQI composites 2, 4, 6 and 7 as defined in Buysse et al. (1989). Significance is tested with two‐tailed, unpaired Student's *t*‐tests (uncorrected *p*‐values). The upper and lower edge of the box represent first and third quartiles, the median value is represented by the line within the box and whiskers extend from the edge of the box to 1.5 times the interquartile range.
**FIGURE S6.** Global Pittsburgh Sleep Quality Index (PSQI) score and age‐adjusted cognitive function total composite (CFTC) score relative to circadian time. PSQI score (a) and CFTC score (b) relative to circadian time of MR‐acquisition. Vertical jitter was added in (a) in order to visualize overlaying datapoints. Correlations are tested with Pearson correlation tests (uncorrected *p*‐values).
**FIGURE S7.** Age‐adjusted cognitive function total composite (CFTC) score relative to global Pittsburgh Sleep Quality Index (PSQI) score. Horizontal jitter was added in order to visualize overlaying datapoints. Correlations are tested with Pearson correlation tests (uncorrected *p*‐values).
**TABLE S1.** Regional microstructural measures relative to circadian time.
Per region correlations coefficients between circadian time of acquisition and extra‐neurite mean diffusivity (exMD) and intra‐neurite volume fraction (INVF), respectively, with corresponding *p*‐values (Bonferroni corrected at *α* = 0.05). For exMD, correlations were tested with Pearson's correlation test. For INVF, correlation with circadian time of acquisition was tested in a linear model after correction for BMI, age and Pittsburgh Sleep Quality Index (PSQI) score. Statistically significant results are highlighted in bold.

## Data Availability

Data used in this study is publicly available at https://www.humanconnectome.org/.
