# Supplementary material for: Perivascular space diffusivity and brain microstructural measures are associated with circadian time and sleep quality
Source: J Sleep Res. 2024 Apr 27;33(6):e14226. doi: 10.1111/jsr.14226 (PMC11512690; doi:10.1111/jsr.14226)
Supplement: Supplementary file 1 — FIGURE S1. Measures included in the analysis relative to release package. (a) Diffusion tensor image analysis along the perivascular space (DTI‐ALPS) index. (b) Average grey matter intra‐neurite volume fraction (INVF). (c) Average grey matter extra‐neurite mean diffusivity (exMD). (d) Average white matter fractional anisotropy (FA). The upper and lower edge of the box represent first and third quartiles, the median value is represented by the line within the box, and whiskers extend from the edge of the box to 1.5 times the interquartile range. FIGURE S2. Average white matter fractional anisotropy (FA) relative to circadian time. Average white matter FA relative to circadian time across subjects with a calculated circadian time above zero. Correlation is tested with a Pearson correlation test. FIGURE S3. Per region Pearson correlation coefficients between subject age and intra‐neurite volume fraction (INVF). FIGURE S4. Regional microstructural measures relative to circadian time. (a) Per region β coefficients from a linear model correlating average grey matter intra‐neurite volume fraction (INVF) with circadian time of acquisition after correction for BMI, age and Pittsburgh Sleep Quality Index score. (b) Per region Pearson correlation coefficients between circadian time of acquisition and extra‐neurite mean diffusivity (exMD). Corresponding statistics can be found in Table S1. FIGURE S5. Average grey matter intra‐neurite volume fraction (INVF) relative to Pittsburgh Sleep Quality Index (PSQI) composite scores without correction for BMI and age. Average grey matter INVF relative to PSQI composites 2, 4, 6 and 7 as defined in Buysse et al. (1989). Significance is tested with two‐tailed, unpaired Student's t‐tests (uncorrected p‐values). The upper and lower edge of the box represent first and third quartiles, the median value is represented by the line within the box and whiskers extend from the edge of the box to 1.5 times the interquartile range. FIGURE S6. Global P [file JSR-33-e14226-s001.docx]

**Supporting information for:**

**Perivascular space diffusivity and brain microstructural measures are associated with circadian time and sleep quality**

Kristoffer Brendstrup-Brix^1,2^, Sara Marie Ulv Larsen^1,2^, Hong-Hsi Lee^3,4^, Gitte Moos Knudsen^1,2^

^1^Neurobiology Research Unit, Copenhagen University Hospital Rigshospitalet, Copenhagen, Denmark

^2^Faculty of Health and Medical Sciences, University of Copenhagen, Copenhagen, Denmark

^3^Athinoula A. Martinos Center for Biomedical Imaging, Department of Radiology, Massachusetts General Hospital, Charlestown, MA 02129, USA

^4^Harvard Medical School, Boston, MA 02115, USA


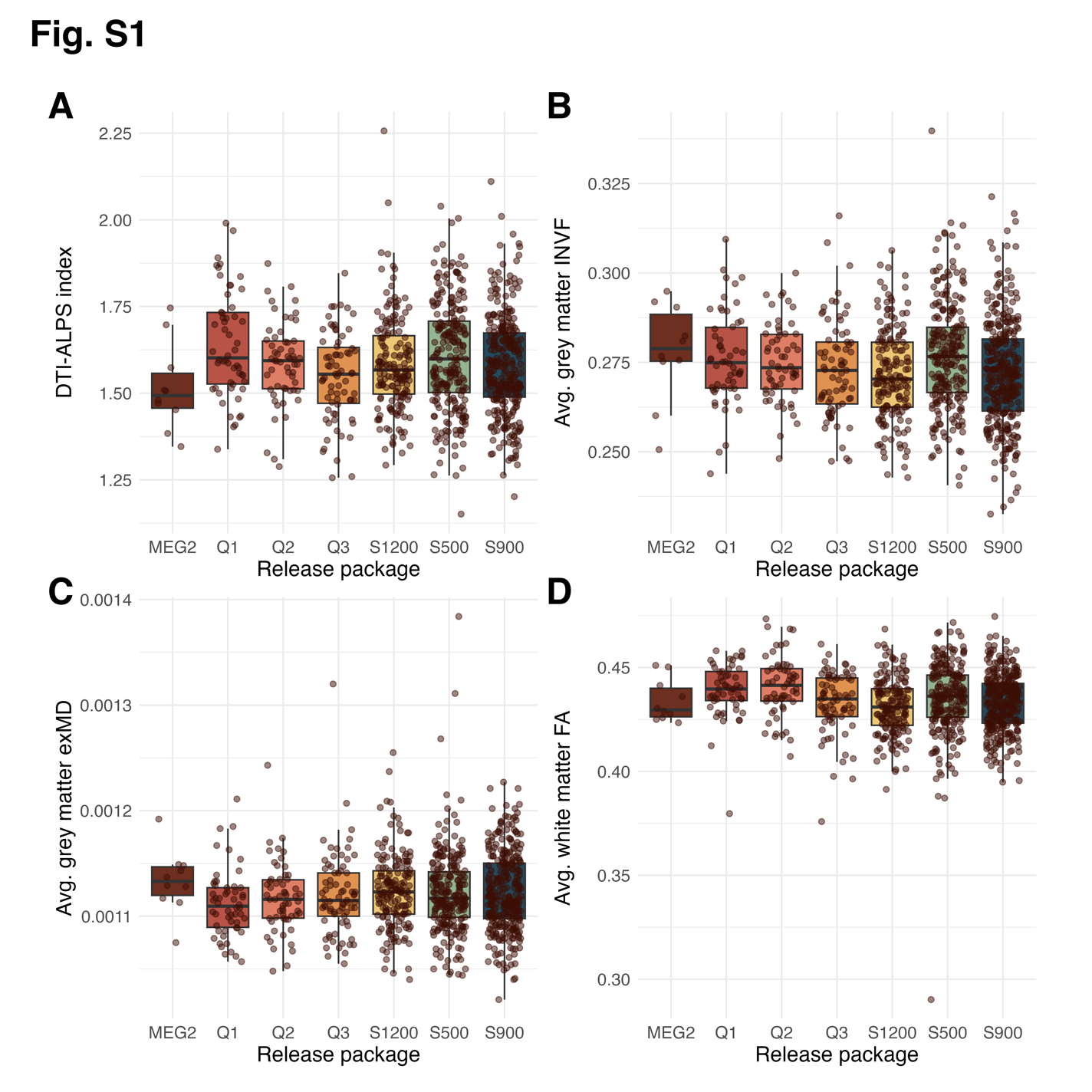


**Figure S1:** **Measures included in the analysis relative to release package.** **A** Diffusion tensor image analysis along the perivascular space (DTI-ALPS) index. **B** Average grey matter intra-neurite volume fraction (INVF). **C** Average grey matter extra-neurite mean diffusivity (exMD). **D** Average white matter fractional anisotropy (FA). The upper and lower edge of the box represent first and third quartiles, the median value is represented by the line within the box, and whiskers extend from the edge of the box to 1.5 times the interquartile range.


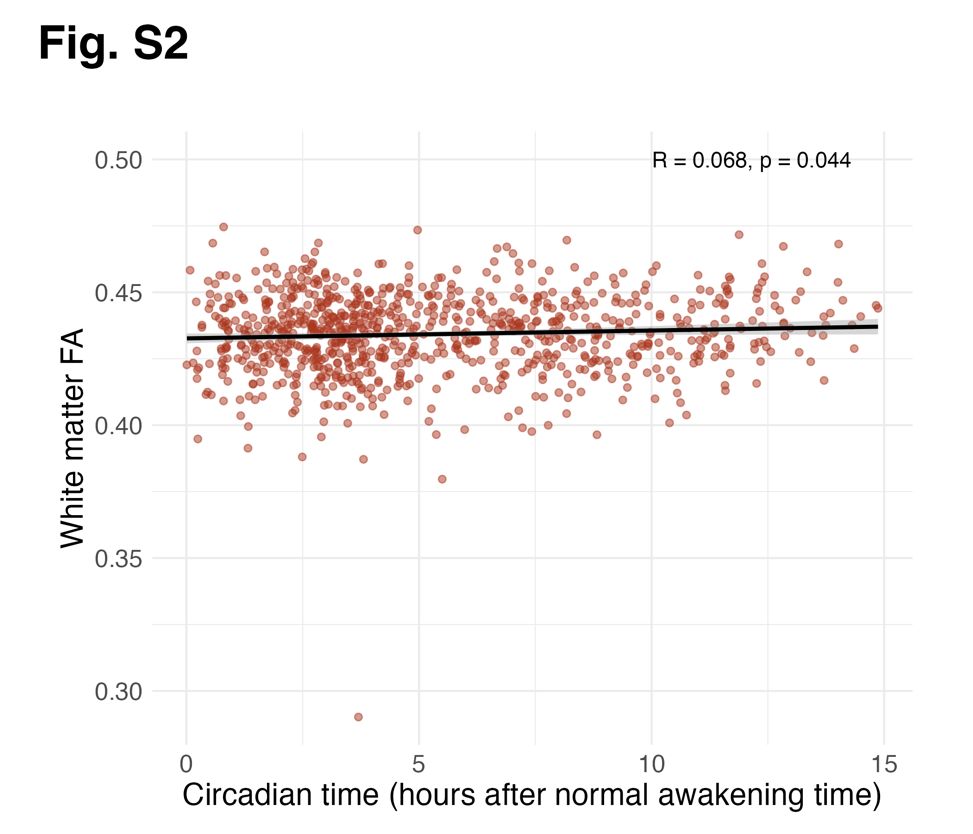


**Figure S2:** **Average white matter fractional anisotropy (FA) relative to circadian time.** Average white matter FA relative to circadian time across subjects with a calculated circadian time above zero. Correlation is tested with a Pearson correlation test.

*
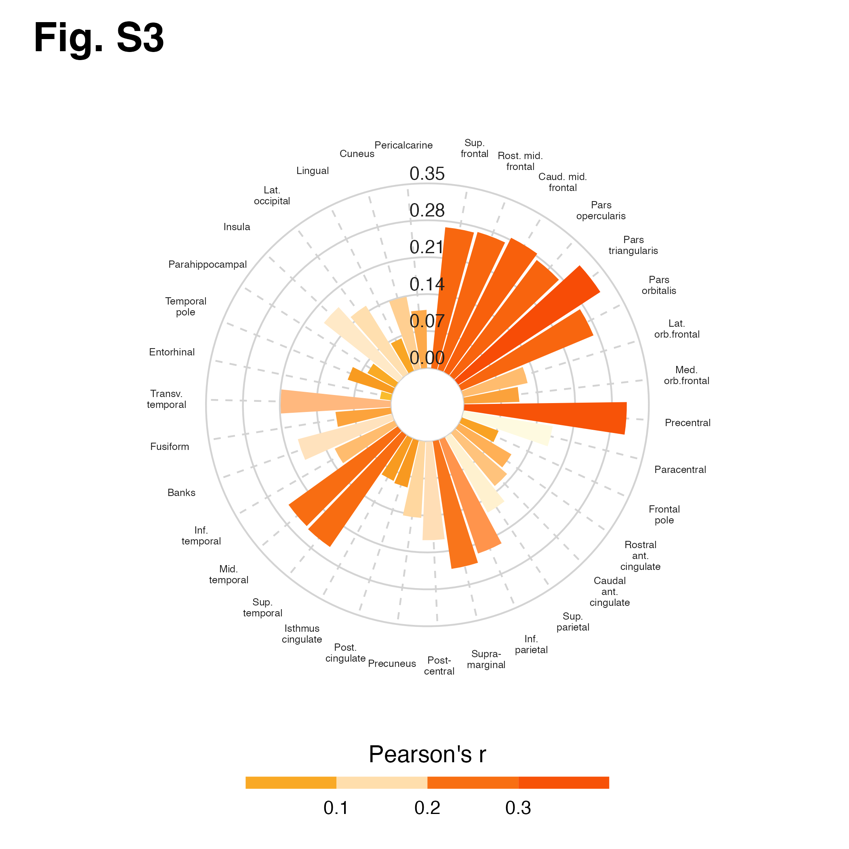
*

**Figure S3:** **Per region Pearson correlation coefficients between subject age and intra-neurite volume fraction (INVF).**


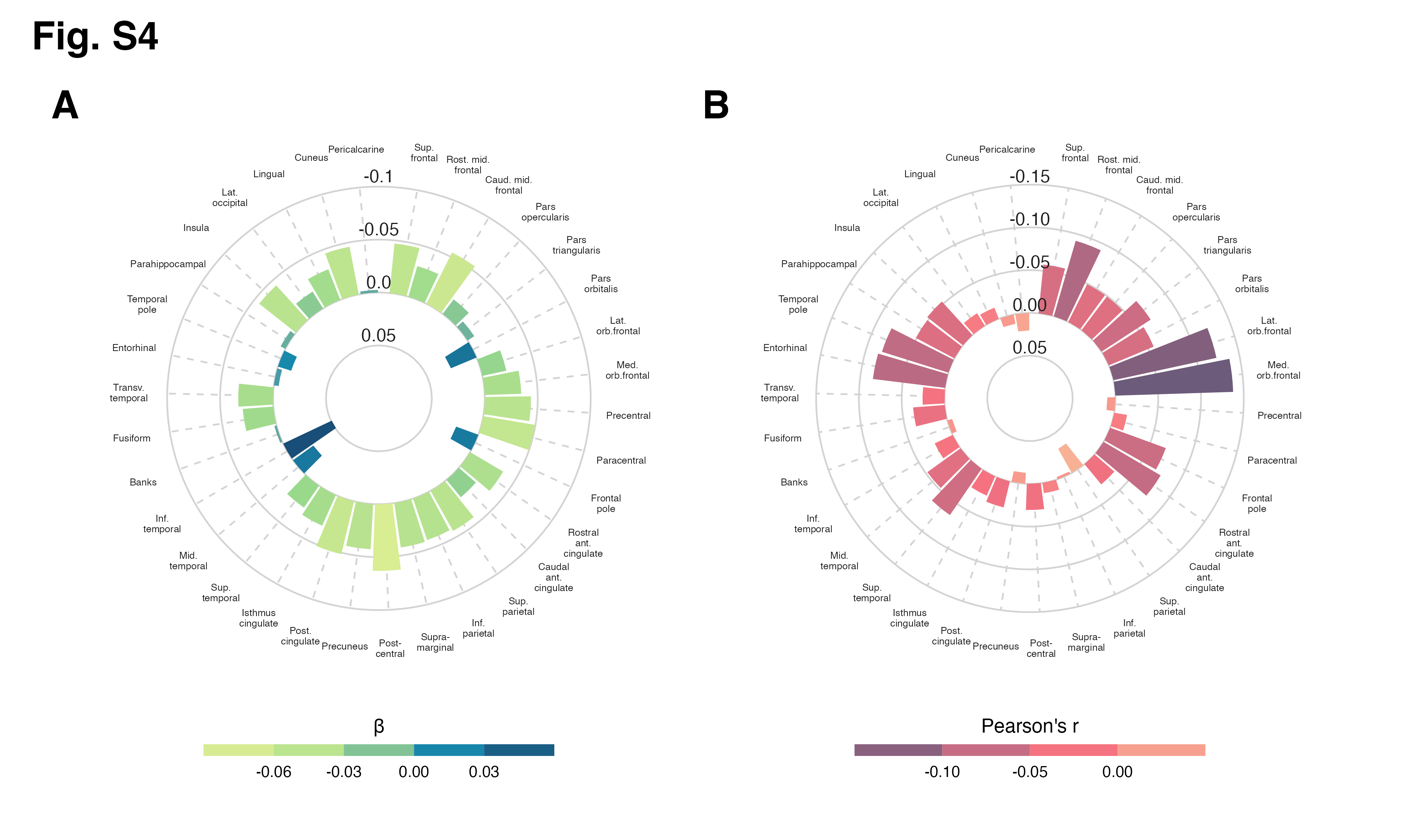
**Figure S4:** **Regional microstructural measures relative to circadian time. A** Per region β coefficients from a linear model correlating average grey matter intra-neurite volume fraction (INVF) with circadian time of acquisition after correction for BMI, age, and Pittsburg Sleep Quality Index score. **B** Per region Pearson correlation coefficients between circadian time of acquisition and extra-neurite mean diffusivity (exMD). Corresponding statistics can be found in table S1.


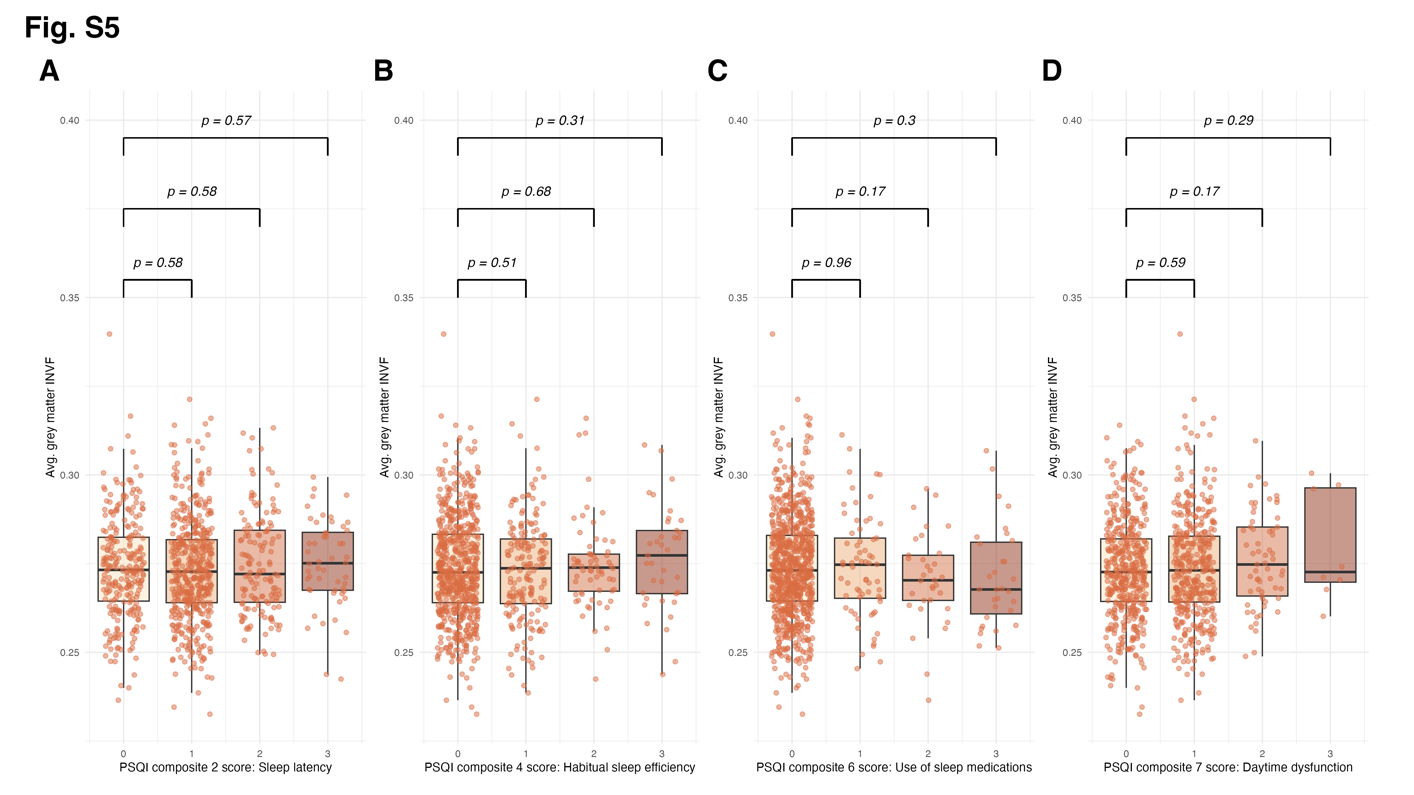
**Figure S5: Average grey matter intra-neurite volume fraction (INVF) relative to Pittsburg Sleep Quality Index (PSQI) composite scores without correction for BMI and age.** Average grey matter INVF relative to PSQI composites 2, 4, 6, and 7 as defined in Buysse et al. (33). Significance is tested with two-tailed, unpaired Student’s t-tests (uncorrected p values). The upper and lower edge of the box represent first and third quartiles, the median value is represented by the line within the box, and whiskers extend from the edge of the box to 1.5 times the interquartile range.


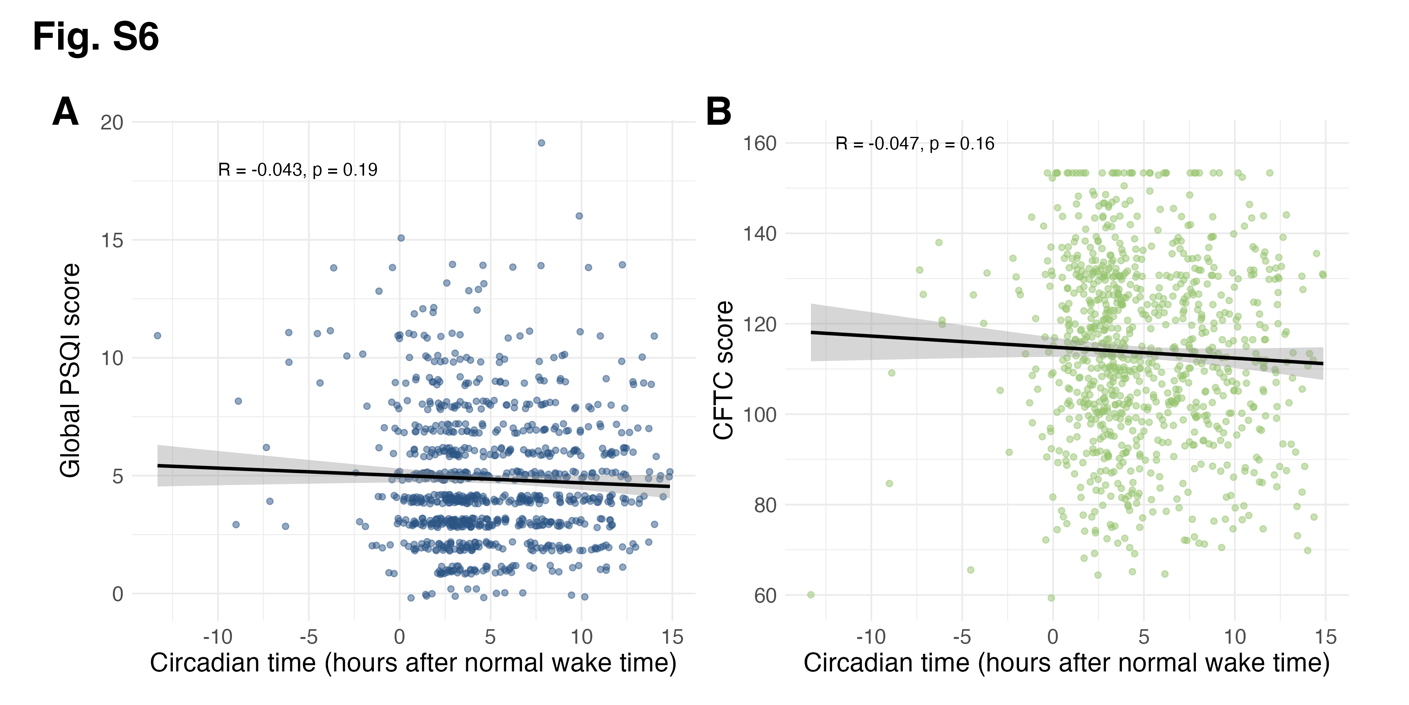
**Figure S6: Global Pittsburg Sleep Quality Index (PSQI) score and age-adjusted Cognitive Function Composite (CFTC) score relative to circadian time.** PSQI score (**A**) and CFTC score (**B**) relative to circadian time of MR-acquisition. Vertical jitter was added in A in order to visualize overlaying datapoints. Correlations are tested with Pearson correlation tests (uncorrected p values).


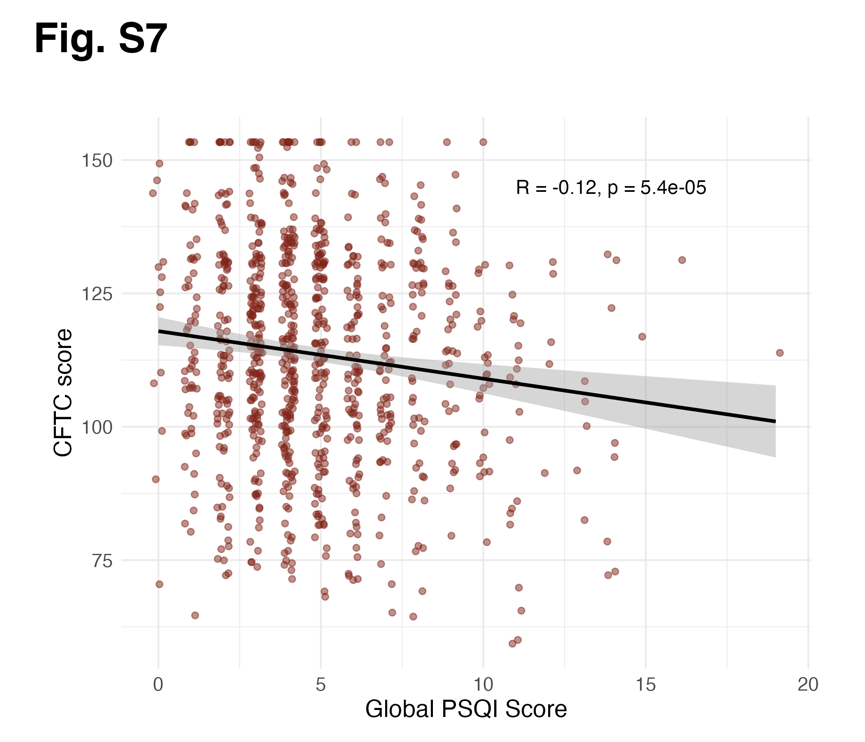


**Figure S7:** **Age-adjusted Cognitive Function Composite (CFTC) score relative to global Pittsburg Sleep Quality Index (PSQI) score.** Horizontal jitter was added in order to visualize overlaying datapoints. Correlations are tested with Pearson correlation tests (uncorrected p values).

|  | exMD,  Pearson’s *r* | exMD, *p* | exMD,  *p_FWER_* | INVF,  *β* | INVF, *p* | INVF, *p_FWER_* |
| --- | --- | --- | --- | --- | --- | --- |
| Banks STS | 0.013 | 0.70 | 1.00 | -0.002 | 0.95 | 1.00 |
| Caud. mid. frontal | -0.028 | 0.40 | 1.00 | -0.054 | 0.09 | 1.00 |
| Caudal ant. cingulate | -0.045 | 0.18 | 1.00 | -0.021 | 0.51 | 1.00 |
| Cuneus | 0.016 | 0.63 | 1.00 | -0.046 | 0.14 | 1.00 |
| Entorhinal | -0.082 | 0.01 | 0.46 | 0.005 | 0.89 | 1.00 |
| Frontal pole | -0.059 | 0.08 | 1.00 | 0.022 | 0.50 | 1.00 |
| Fusiform | -0.031 | 0.35 | 1.00 | -0.029 | 0.35 | 1.00 |
| Inf. parietal | 0.009 | 0.80 | 1.00 | -0.041 | 0.19 | 1.00 |
| Inf. temporal | -0.015 | 0.65 | 1.00 | 0.051 | 0.12 | 1.00 |
| Insula | -0.048 | 0.15 | 1.00 | -0.044 | 0.17 | 1.00 |
| Isthmus cingulate | -0.025 | 0.45 | 1.00 | -0.032 | 0.32 | 1.00 |
| Lat. occipital | -0.011 | 0.73 | 1.00 | -0.019 | 0.55 | 1.00 |
| Lat. orb.frontal | **-0.113** | **6 · 10^-4^** | **0.02** | -0.023 | 0.49 | 1.00 |
| Lingual | -0.013 | 0.69 | 1.00 | -0.031 | 0.31 | 1.00 |
| Med. orb.frontal | **-0.132** | **5 · 10^-5^** | **0.002** | -0.034 | 0.30 | 1.00 |
| Mid. temporal | -0.041 | 0.22 | 1.00 | 0.024 | 0.45 | 1.00 |
| Paracentral | -0.011 | 0.74 | 1.00 | -0.050 | 0.13 | 1.00 |
| Parahippocampal | -0.053 | 0.11 | 1.00 | -0.005 | 0.87 | 1.00 |
| Pars opercularis | -0.046 | 0.16 | 1.00 | -0.017 | 0.59 | 1.00 |
| Pars orbitalis | -0.048 | 0.15 | 1.00 | 0.026 | 0.41 | 1.00 |
| Pars triangularis | -0.065 | 0.05 | 1.00 | -0.008 | 0.81 | 1.00 |
| Pericalcarine | 0.024 | 0.46 | 1.00 | -0.002 | 0.94 | 1.00 |
| Post. cingulate | -0.026 | 0.43 | 1.00 | -0.051 | 0.12 | 1.00 |
| Postcentral | -0.029 | 0.38 | 1.00 | -0.063 | 0.05 | 1.00 |
| Precentral | 0.015 | 0.66 | 1.00 | -0.044 | 0.16 | 1.00 |
| Precuneus | 0.018 | 0.60 | 1.00 | -0.043 | 0.18 | 1.00 |
| Rostral ant. cingulate | -0.072 | 0.03 | 0.96 | -0.030 | 0.35 | 1.00 |
| Rostral middle frontal | -0.086 | 0.01 | 0.30 | -0.036 | 0.27 | 1.00 |
| Sup. frontal | -0.052 | 0.11 | 1.00 | -0.047 | 0.14 | 1.00 |
| Sup. parietal | 0.036 | 0.28 | 1.00 | -0.043 | 0.18 | 1.00 |
| Sup. temporal | -0.058 | 0.08 | 1.00 | -0.025 | 0.42 | 1.00 |
| Supramarginal | -0.008 | 0.80 | 1.00 | -0.042 | 0.18 | 1.00 |
| Temporal pole | -0.079 | 0.02 | 0.59 | 0.013 | 0.69 | 1.00 |
| Transverse temporal | -0.020 | 0.54 | 1.00 | -0.033 | 0.30 | 1.00 |

**Table S1:** **Regional microstructural measures relative to circadian time**. Per region correlations coefficients between circadian time of acquisition and extra-neurite mean diffusivity (exMD) and intra-neurite volume fraction (INVF), respectively, with corresponding p values (Bonferroni corrected at α = .05). For exMD, correlations were tested with Pearson’s correlation test. For INVF, correlation with circadian time of acquisition was tested in a linear model after correction for BMI, age, and Pittsburg Sleep Quality Index (PSQI) score. Statistically significant results are highlighted in bold.
